# The Composition and Function of Pigeon Milk Microbiota Transmitted From Parent Pigeons to Squabs

**DOI:** 10.3389/fmicb.2020.01789

**Published:** 2020-08-04

**Authors:** Jinmei Ding, Nan Liao, Yuming Zheng, Lingyu Yang, Hao Zhou, Ke Xu, Chengxiao Han, Huaixi Luo, Chao Qin, Chunhong Tang, Longxing Wei, He Meng

**Affiliations:** ^1^Shanghai Key Laboratory of Veterinary Biotechnology, Department of Animal Science, School of Agriculture and Biology, Shanghai Jiao Tong University, Shanghai, China; ^2^Shanghai Xinrong Big Emperor Pigeon Breeding Professional Cooperation, Shanghai, China; ^3^Fengxian District Animal Disease Prevention and Control Center, Shanghai, China

**Keywords:** pigeon milk, microbiota, squabs, parent pigeons, composition, function, transmitted

## Abstract

Mammalian neonates obtain antibodies, nutrients, and microbiota from breast milk that help them resist the complex growth environment. Similar to mammals’ lactation behavior for their offspring, parent pigeons regurgitate pigeon milk (PM) from their crops to feed the squabs. Whether pigeon milk is as valuable as mammalian milk is not clear, especially in terms of microbiota. This study adopted 16S rRNA gene sequencing to investigate the microbial composition and function in pigeon milk. We found abundant microbiota in pigeon milk. The dominant genera in parent pigeons’ milk were *Lactobacillus*, *Enterococcus*, *Veillonella*, and *Bifidobacterium*. An analysis of squab milk (SM) showed that *Lactobacillus* also accounted for a considerable proportion, followed by *Bifidobacterium*. Most of the squab milk microbial genera were also detected in parent pigeons. Microbial functional analysis showed that the squab milk microbes were more involved in the pathways of carbohydrate metabolism, amino acid metabolism, and energy metabolism. These findings indicated that microbiota play an important role in squabs and can be transmitted from parent pigeons to squabs by pigeon milk. The presence of plentiful probiotics in squabs also suggests that adding probiotics in artificial pigeon milk may promote the growth and development of squabs and improve the production performance of pigeons.

## Introduction

Pigeon, the common name for birds of the taxonomic family Columbidae and the order Columbiformes, is an essential economic animal that provides meat and eggs for humans. After fertilized eggs are incubated for 28 days, the squabs hatch. Pigeons are altricial, meaning that newly-hatched squabs are unable to feed independently; they must be fed “pigeon milk” (PM) in a mouth-to-mouth manner to survive. For most poultry, the crop plays the role of temporary food storage, but in pigeons, the crop acts as an organ that produces pigeon milk for squabs, in addition to storing food (Gillespie et al., 2011). Just as mammals lactate for their offspring, parent pigeons regurgitate pigeon milk from their crops to feed the squabs (Luo et al., 2017). Unlike mammals, both male and female pigeons produce pigeon milk (Gillespie et al., 2012). Pigeon milk contains protein (60%), fat (32–36%), carbohydrate (1–3%), minerals (calcium, potassium, sodium, and phosphorus), and antibodies (Davies, 1939; Kocianova et al., 1993). Up to 7 days of age, squabs mainly rely on pigeon milk to obtain nutrients, while between 8 and 14 days of age, the pigeon milk includes a large amount of food initially digested by their parents (Horseman and Buntin, 1995). Because of this special feeding pattern, the number of pigeon’s offspring and their survival rate are very low, which makes it difficult for the pigeon industry to achieve intensive breeding. Although researchers want to improve the industrialization of pigeons by producing artificial pigeon milk, the components and values of artificial pigeon milk are still limited because most of the studies only focused on the nutrition and immune function of pigeon milk (Goudswaard et al., 1979; Shetty et al., 1994). Therefore, the efficiency of the pigeon breeding industry remains low due to poor understanding of pigeon milk composition, especially regarding the microbiota in pigeon milk (Shetty et al., 1990).

There is a symbiotic relationship between microbiota and their hosts (Rees et al., 2018; Dietz et al., 2019). The main benefit of microbes was to obtain a relatively stable habitat and adequate food source (Kohl, 2012; McFall-Ngai et al., 2013). Meanwhile, microbes play an important role in many aspects of host physiology, including nutrition, metabolism, and intestinal homeostasis (Walker et al., 2017). Early colonization of microbiota can have long-standing consequences on host such as determining the production of essential metabolites which facilitate postnatal development and enhance immune function (Lee and Mazmanian, 2010; Funkhouser and Bordenstein, 2013; Gensollen et al., 2016; Stinson et al., 2017). Neonates of mammals can acquire maternal microbiota through the placenta, amniotic fluid, vagina, and breast milk (DiGiulio et al., 2008; Satokari et al., 2009; Albesharat et al., 2011; Stout et al., 2013; Aagaard et al., 2014). The prenatal exposure is an important step in modulating the embryonic development and the maturation of immune system (Nylund et al., 2014). Fetuses are highly susceptible to disease infections, not only because their immature immune system is less capable of generating adaptive immune effectors, such as antibodies, but also because they lack diverse commensalmicrobiota that can antagonize pathogens independently of host responses (Basha et al., 2014; Simon et al., 2015; Zheng et al., 2020). Although the chicken embryo is isolated from the mother, the core microbial colonizers of maternal hens can be transmitted to the embryos in the process of fertilization and egg formation in the oviduct (Ding et al., 2017). Likewise, prenatal bacteria transfer may occur in other birds. The relatively high percentage of shared operational taxonomic units (OTUs) between neonates and females is a strong indication that neonates of rock pigeons obtain bacteria through prenatal transfer (Dietz et al., 2019). Research has shown that lactobacilli is important in maintaining a healthy microbial balance in the chicken crop (Fuller, 1977), but as regard to crop secretions, it is not known the pigeonmilkmicrobial composition and function, and whether these microbes can be transmitted from parent pigeons to squabs.

In this study, we adopted new generation high throughput sequencing technology to analyze the composition and function of microbiota in pigeon milk and pigeon intestines at different developmental stages.

## Materials and Methods

### Animal and Sample Collection

**Figure 1:**
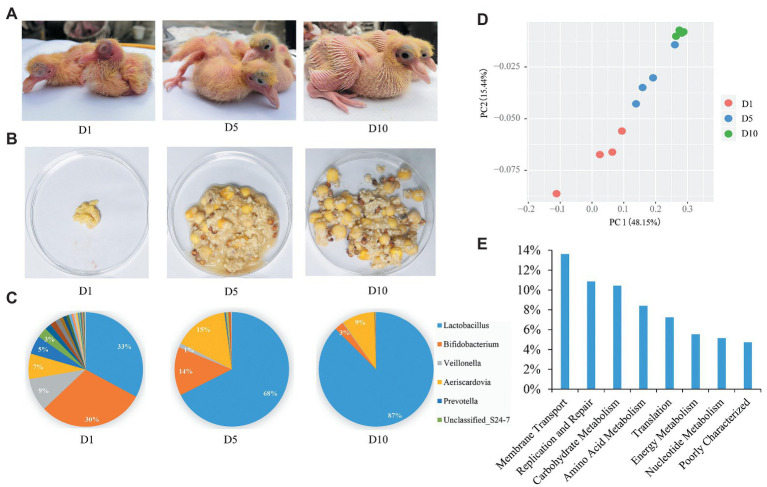
Aggregate squab milk microbiota composition and function. **(A)** The squabs at 1-day (D1), 5-day (D5), and 10-day (D10). **(B)** Morphology of squab milk at different developmental stages. **(C)** Distribution of the squab milk microbiota among different developmental stages at the genus level. Only major taxonomic groups are shown. **(D)** Dynamic distribution of squab milk microbiota at different developmental stages shown by principal component analysis (PCA) plot. **(E)** The functional pathways of squab milk microbiota. Only major pathways are shown.

The pigeons to be sampled were selected from the Shanghai Xinrong Big Emperor Pigeon Breeding Professional Cooperative. A total of 24 pigeons were selected, including 12 parent pigeons who were 2 years old and having similar weight and size, and 12 squabs of different ages [4 each at 1-day (D1), 5-day (D5), and 10-day (D10) of age; Figure 1A and Supplementary Figure 1A]. Samples of parent and squab pigeons were collected on the same day (Figure 1B and Supplementary Figure 1B). In order to reduce the impact of environmental factors and verify its effectiveness, we conducted effective experimental controls on the management and feeding of the pigeons, as manifested by the fact that all individuals were kept under the same roof, the parent pigeons are raised in the same cage with their offspring, and the feed formula of the parent pigeons was consistent. Routine feeding procedures were followed for feeding management, and the subjects had free access to food and water. None of the selected pigeons had been exposed to antibiotics within a month. All experiments on these animals were conducted in accordance with the animal welfare protection provisions of the Shanghai Jiao Tong University. We collected 48 samples in total, which consisted of 12 parent pigeon milk samples and 12 gut content (PG) samples from parent pigeons, 12 crop content samples [referred to as “squab milk” (SM)] and 12 gut content (SG) samples from squabs. Pigeon milk and gut content were collected individually with sterile tweezers and were placed into sterile centrifuge tube. The procedures of sample collection and subsequent operation were carried out on a clean bench under aseptic conditions. The samples were immediately stored at −80°C after collection.

### DNA Extraction and 16S rRNA Gene Sequencing

The TIANGEN DNA stool mini kit (TIANGEN, cat#DP328) was used for microbial genome DNA extraction from pigeon milk and gut content samples, following the manufacturer’s instructions. The DNA quantity and quality were assessed by a Nanodrop spectrophotometer (Thermo scientific, United States). The V3–V4 hypervariable region of the 16S rRNA gene was amplified by PCR using sample-specific sequence barcoded fusion primers: forward primer 338F (5'-ACTCCTACGGGAGGCAGCA-3'), and reverse primer 806R (5'‐ GGACTACHVGGGTWTCTAAT-3'). The PCR reaction conditions and product purification were performed as previous publication (Zhao et al., 2013). 16S rRNA gene sequencing of 48 samples was carried out using Illumina MiSeq (Illumina, United States) by the Shanghai Personal Biotechnology Limited Company, Shanghai, China. Our sequence reads quality control criteria were as follows: the reads with mean quality higher than 30, no ambiguous bases, sequence length longer than 150 bp, no chimeras, no adaptor contaminations, and no host contaminating. The genome was assembled by the filtered sequences according to the overlap longer than 10 bp between read 1 and read 2 and without mismatches. Trimmed sequences were uploaded to QIIME for further analysis. The DNA sequences are publicly available in Metagenome Rapid Annotation using Subsystem Technology (MG-RAST) under the project name “pigeon-milk-microbiota”^1^.

### Taxonomy Classification and Statistical Analysis

Using QIIME V.1.9.1, we merged, applied quality control, and clustered the 16S rRNA gene reads into OTUs. Taxonomic groups were based on the GreenGene Database V.13_8 using closed references to perform reference-based OTU clustering (Edgar, 2010; McDonald et al., 2012). OTUs that were present in at least 12 samples were used for the next step. The OTU abundance counts were log2 transformed and normalized by subtracting the mean of all transformed values and dividing by the standard deviation of all log-transformed values for the given sample. In the end, the abundance profiles for 48 samples exhibited a mean of 0 and a standard deviation of 1. Normalized abundance was used to perform statistical analyses. Values employed for alpha diversity (Chao1 index, Shannon index, and Simpson index) and beta diversity [non-metric multidimensional scaling (NMDS; weighted UniFrac distance metrics) and principle component analysis (PCA)] were generated by QIIME V.1.9.1.^2^ The Venn diagrams were generated using mothur (Schloss et al., 2009). Box plots and bar charts were created by SigmaPlot (Kornbrot, 2000). Two-side Welch’s *t*-test and multiple comparisons were applied to identify different taxa microbes among groups. All values of *p* were adjusted using the Benjamini–Hochberg method. In the figures and tables, *p* < 0.05 indicates statistical significance (^*^p < 0.05, ^**^p < 0.01; Benjamini and Hochberg, 1995). Statistical analyses and data visualization were performed using R V.3.5.0 (under RStudio V.1.1.453; Dessau and Pipper, 2008) and STAMP (Parks and Beiko, 2010). Microbial functions were predicted using 16S rRNA gene sequence data by PICRUSt (Langille et al., 2013). The OTUs were mapped to gg13.5 database at 97% similarity by QIIME’s command “pick_closed_otus.” The OTUs abundance was normalized automatically using 16S rRNA gene copy numbers from known bacterial genomes in integrated microbial genomes. The predicted genes and their functions were aligned to the Kyoto Encyclopedia of Genes and Genomes (KEGG) database, and differences among groups were compared through software STAMP.

## Results

### The Squab Milk Microbial Characteristics and Dynamic Distribution at Different Developmental Stages

Twelve squab milk samples from the craw content of squabs were collected. As in previous studies (Horseman and Buntin, 1995), we also found that the early milk of squabs is cheesy, and later milk contains undigested food from their parents (Figure 1B and Supplementary Figure 1). A total of 550,696 high quality reads were yielded from 12 squab milk samples by 16S rRNA gene sequencing. On average, 45,891 reads per sample were classified into different taxonomies and diversity analyses. Based on the results of OTUs, 8 phyla, 96 genera, and 114 species of microbiota were recorded. The most abundant phylum was *Firmicutes* (67%), followed by *Actinobacteria* (27%), *Bacteroidetes* (4%), and *Cyanobacteria* (2%) in squab milk (Supplementary Figure 2A). Correspondingly, the dominant microbial genera were *Lactobacillus*, *Bifidobacterium*, *Aeriscardovia*, and *Veillonella* (Figure 1C). Beta diversity suggested the dynamic changes of these microbes in the squab milk at different developmental stages (D1, D5, and D10; Figure 1D). PCA showed that the microbiota were clustered at similar developmental stages. The phylogenetic distance of the first day microbiota significantly diverged from the 10-day in squab milk, and the 5-day microbial phylogenetic distance was associated with both (Figure 1D). At the genus level, the microbial composition differed among the different developmental stages. As the squab’s development with time, we observed that the proportions of the genera changed. Among the 96 genera, statistical analysis found that 38 of them were conspicuously different with time (*p* < 0.05; Supplementary Table 1). The most abundant genus carried by squab milk was *Lactobacillus*, which showed a significantly increasing trend according to developmental stages (*p* < 0.01). At D1, the percentage of *Lactobacillus* was 33%, which increased to 68% at D5 and to 87% at D10 (Figure 1C). *Unclassified_Streptophyta* also observably raised from 0.05% (D1) to 3.42% (D10) (*p* < 0.01; Supplementary Figure 2B). In contrast, *Bifidobacterium*, *Veillonella*, and *Prevotella* diminished with growth. *Bifidobacterium* decreased from 30% (D1) to 14% (D5) and to 3% (D10; Figure 1C). *Veillonella* and *Prevotella* were also markedly ranged between different developmental stages (*p* < 0.01; Supplementary Table 1). The rate at which the genera increased from D1 to D5, and then had decreased at D10, corresponded with the proportion of *Aeriscardovia* (7, 15, and 9%; Figure 1C). To further investigate the functions of squab milk microbiota, we used PICRUSt to produce predicted microbial functional pathways from 16S rRNA gene sequence data (Figure 1E). From this analysis, we observed that most of the squab milk microbes are involved in the pathways of membrane transport, replication and repair, carbohydrate metabolism, amino acid metabolism, and energy metabolism, which are important to growth and development of the organism (Figure 1E and Supplementary Table 2).

### Microbiota Can Be Transmitted From Parents to Squabs by Pigeon Milk

**Figure 2:**
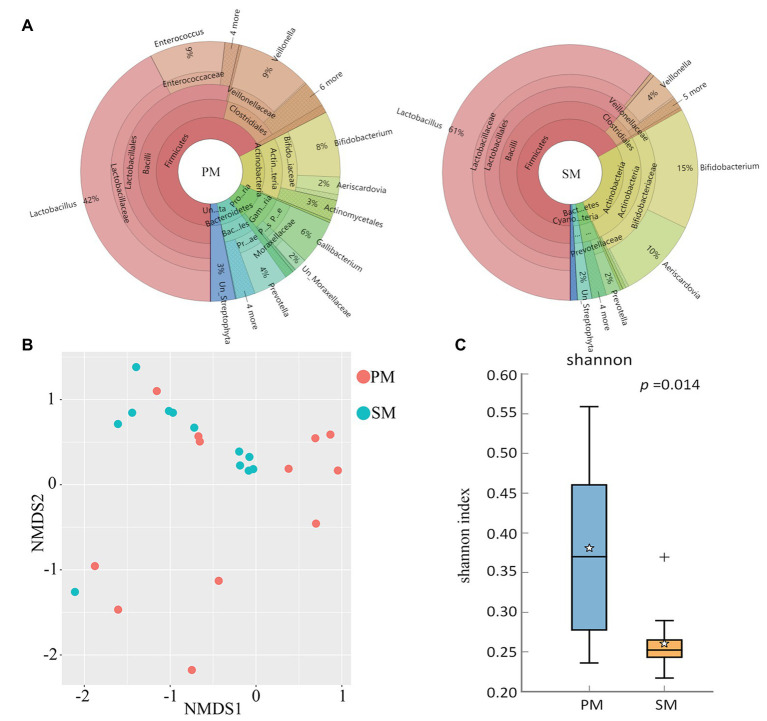
Comparison and statistical analysis of microbiota between parent pigeon milk (PM) and squab milk (SM). **(A)** The distribution of the microbiota for PM and SM. **(B)** Non-metric multidimensional scaling (NMDS; weighted UniFrac distance) plot. **(C)** Alpha diversity analysis by Shannon index.

To explore the origin of pigeon milk microbiota, we surveyed milk microbial composition and diversity between parent pigeon milk and squab milk (Figure 2). Five phyla – *Firmicutes*, *Actinobacteria*, *Proteobacteria*, *Bacteroidetes*, and *Cyanobacteria* – were present as major components in pigeon milk (Figure 2A). The pigeon milk genera in parent pigeons were dominated by *Lactobacillus*, *Enterococcus*, *Veillonella*, and *Bifidobacterium* (42, 9, 9, and 8%, respectively; Figure 2A). Analysis of squab milk showed that *Lactobacillus* also accounted for a considerable proportion, 61%, followed by *Bifidobacterium*, *Aeriscardovia*, and *Veillonella*, at 15, 10, and 4%, respectively (Figure 2A). The increasing trend of *Lactobacillus* and *Unclassified_Streptophyta*, and decreasing trend of *Veillonella* and *Prevotella* in squab milk were consistent with that in parent pigeons among different developmental stages (Figure 1C and Supplementary Figure 1C). The dynamic changes of *Bifidobacterium*, *Entercococcus*, and *Gallibacterium* in squab milk may be also related to the changes of microbiota in parent pigeons with different developmental stages (Supplementary Figures 2B, 1C). We further explored similarities between the microbial communities of squabs’ and parents’ pigeon milk by beta diversity and alpha diversity. Microbial beta diversity of pigeon milk using a NMDS (weighted UniFrac distance) plot showed the characteristics of the squabs and parent pigeons, indicating a difference of microbial communities (Figure 2B). Chao1 index suggested that their microbial richness was similar (Supplementary Figure 3A). Likewise, the Venn diagram indicated that most of milk microbial genera (93%) in squabs were similar to those of the parent pigeons (Supplementary Figure 3B). These results suggested that pigeon milk is rich in microorganisms, and the parents transfer their microbes to the squabs through pigeon milk. While alpha diversity analysis by Shannon index revealed more community diversity in parent pigeons than in squabs (Figure 2C). The abundance of seven bacteria differed between squabs and parent pigeons (*p* < 0.05; Table 1). *Lactobacillus*, *Bifidobacterium*, *Aeriscardovia*, and *Streptobacillus* were markedly plentiful in squabs, while *Enterococcus* and *Gallibacterium* exhibited a higher abundance in parent pigeons. This difference indicated that beneficial bacteria are more likely to colonize and maintain high abundance in squabs.

**Table 1:** Significant differences of pigeon milk microbial genus abundance between PM and SM (*p* < 0.05).

Phylum	Genus	Relative fold change	*p*
*Firmicutes*	*Enterococcus*	1.86	0.010
*Proteobacteria*	*Gallibacterium*	1.74	0.029
*Actinobacteria*	*Unclassified_Actinomycetaceae*	1.35	0.004
*Fusobacteria*	*Streptobacillus*	−1.27	0.025
*Firmicutes*	*Lactobacillus*	−2.01	0.049
*Actinobacteria*	*Bifidobacterium*	−2.97	0.005
*Actinobacteria*	*Aeriscardovia*	−4.30	0.001

+PM > SM; −PM < SM.

### Gut Microbial Characteristics of Parent Pigeons and Squabs

**Figure 3:**
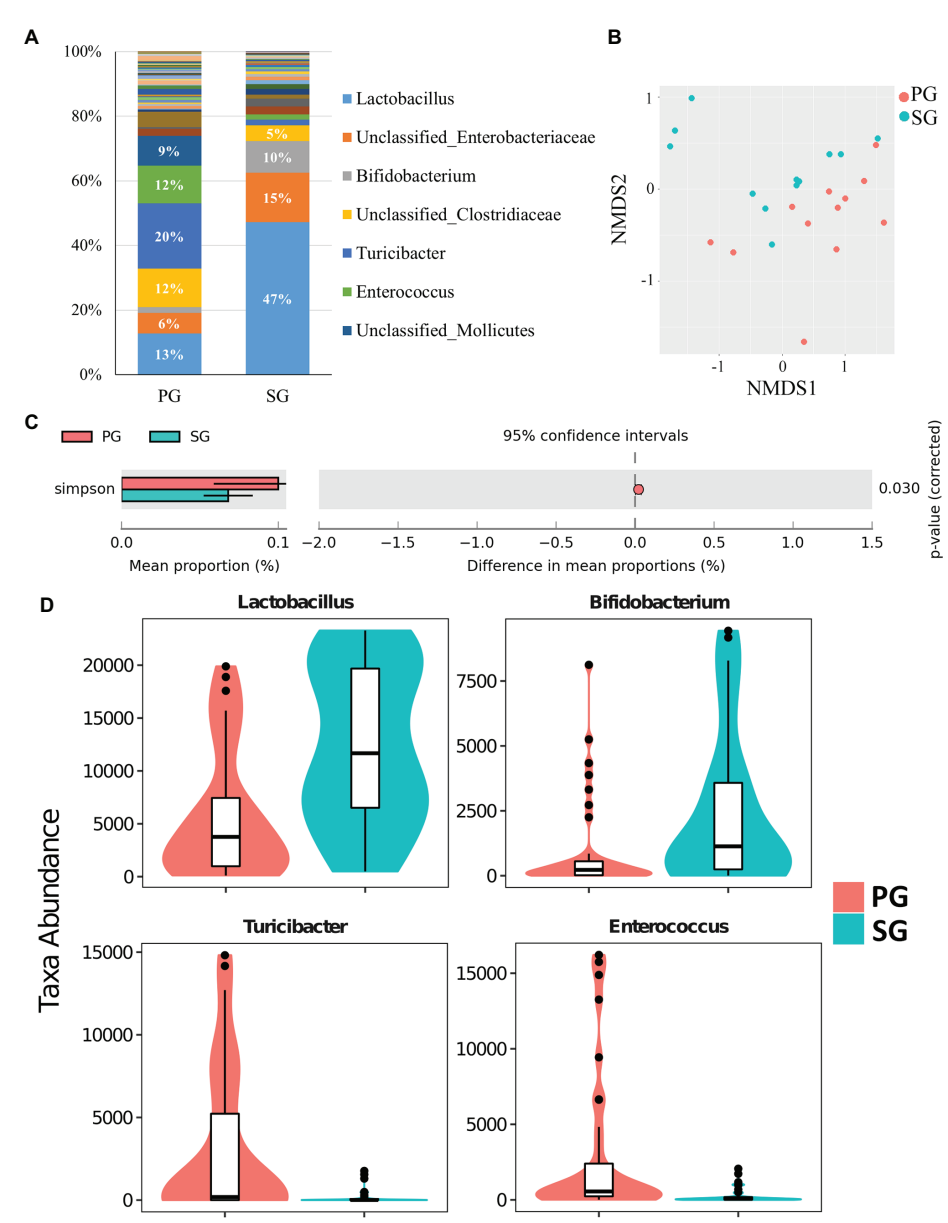
Pigeon gut microbial characteristics and distribution. **(A)** Parent pigeon gut (PG) and squab gut (SG) microbiota composition at the genus level. **(B)** Gut microbial beta diversity of pigeons with a NMDS plot. **(C)** Simpson estimator to exhibit the different community diversities in PG and SG. **(D)** Significantly different gut microbes between PG and SG.

Similar to pigeon milk, the dominant phyla of pigeon gut microbiota were *Firmicutes* (71%), *Actinobacteria* (12%), and *Proteobacteria* (12%; Supplementary Figure 4). We also studied the presence of gut microbiota between parent pigeons (PG) and squabs (SG) at the genus level. The gut communities of parent pigeons were largely dominated by *Turicibacter*, *Lactobacillus*, and *Enterococcus* (Figure 3A). As expected, other bacteria made up a relatively small fraction of the overall community, with *Lactobacillus* (47%) and *Bifidobacterium* (10%) being the prevalent beneficial bacteria in squabs’ gut (Figure 3A). NMDS based on weighted UniFrac distance revealed a significant separation of samples, indicating that the gut microbial communities of parent pigeons and squabs are different (Figure 3B). The gut community diversity was reflected by a reduction of the Simpson index from parent pigeons to squabs (Figure 3C). The significant differences in community structure were also evident from the relative proportion of different taxa across the groups (*p* < 0.05; Supplementary Table 3). Eight genera showed markedly different between the gut microbiota of parent pigeons and squabs, with a clear increase in the relative abundance of *Lactobacillus* and *Bifidobacterium*, and a reduction in *Turicibacter* and *Enterococcus* in squabs, relative to parent pigeons (Figure 3D). The significant difference of gut microbiota between parent pigeons and squabs may be demonstrated that the intestinal microbial structure of squabs was affected by the squab milk microbiota.

### The Comparison of Microbial Composition and Function Between Parent Pigeon Milk and Gut

**Figure 4:**
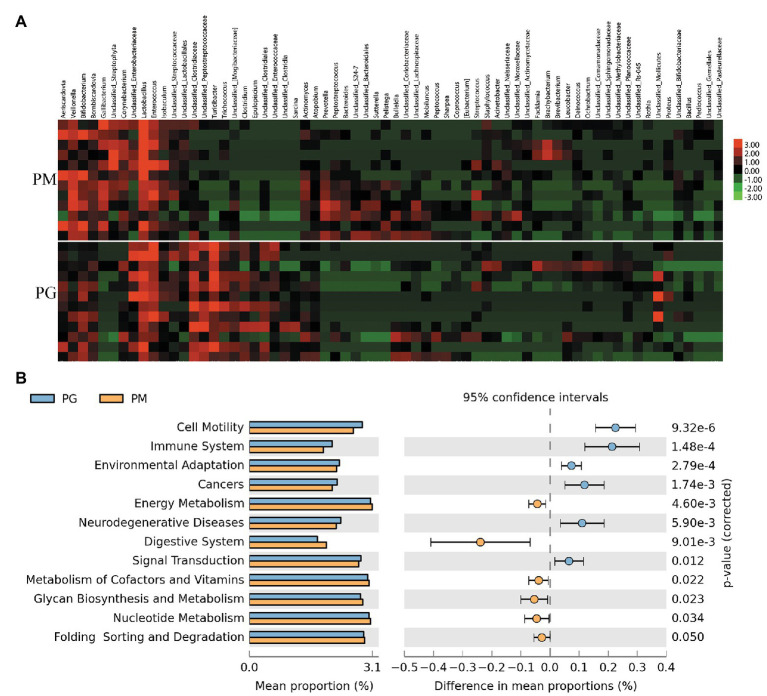
The analysis of the gut microbiota (PG) and the milk microbiota (PM) in parent pigeons. **(A)** Heatmap of hierarchy cluster results for the microbiota of PG and PM at the genus level. **(B)** Significant differences of microbial metabolic pathways for PG and PM.

In order to investigate which microbes of parent pigeons will be transmitted to their progeny, we compared the gut microbiota (PG) and the milk microbiota (PM) in parent pigeons. The phyla of *Firmicutes*, *Proteobacteria*, and *Actinobacteria* were common in PG and PM (Supplementary Figure 5). But the *Bacteroidetes* and *Cyanobacteria* are plentiful in pigeon milk, and *Tenericutes* was the dominant microbial phylum of the pigeon gut. At the genus level, 12 bacteria genera showed a significant difference between the pigeon milk microbiota and gut microbiota (*p* < 0.05; Supplementary Table 4 and Figure 4A). The predominant genera in pigeon milk were *Lactobacillus* (42%), *Enterococcus* (9%), *Veillonella* (9%), and *Bifidobacterium* (8%), while the pigeon gut was dominated by *Turicibacter* (20%), *Lactobacillus* (13%), *Unclassified_Clostridiaceae* (12%), *Enterococcus* (12%), and *Unclassified_Mollicates* (9%; Supplementary Figure 6). The abundances of *Gallibacterium*, *Veillonella*, and *Lactobacillus* in pigeon milk were nearly seven-, four-, and two-fold higher than that in pigeon gut, respectively (Supplementary Table 4). However, some bacteria associated with inflammation, such as *Turicibacter* and *Clostridium* remarkably enriched in pigeon gut microbiota (Supplementary Table 4). Faced with a complex microbial structure, parent pigeons may select some beneficial and valuable microbes to transfer to the squabs. Moreover, based on the analysis of the microbial 16S rRNA gene sequencing data, we discovered that the abundant microbes in pigeon milk were frequently involved in the functional pathways of energy metabolism, digestive system, metabolism of cofactors and vitamins, glycan biosynthesis and metabolism, nucleotide metabolism, and so on (Figure 4B). Compared with pigeon milk, the microbial pathways of immune system, environmental adaptation, and neurodegenerative diseases were abundant in the pigeon gut (Figure 4B).

## Discussion

Pigeon is one of the few birds capable of regurgitating pigeon milk to nourish young squabs, which cannot eat independently like other poultry due to their late maturity. Similar to mammalian breast milk (Boix-Amorós et al., 2016), pigeon milk is highly nutritious, consisting of protein, fat, carbohydrates, and minerals (Xie et al., 2019). Interestingly, we found a mass of microbiota in pigeon milk in this study (Figure 2A). Moreover, the pigeon milk microbiota can be transmitted from parents to squabs. It implies that pigeons not only transfer nutrients, but also microbiota to squabs by pigeon milk to help them cope with the complex living environment. Analyzing the microbial KEGG pathway suggested that galactose, starch, and sucrose metabolism belonging to carbohydrate metabolism were observably higher in squab milk than in parent pigeon milk (Supplementary Table 5). Galactose is a key source of energy and particularly important for early human development (Coelho et al., 2015). The galactose and sucrose metabolism present in milk is a determinant factor in neonatal host defense and inflammatory processes due to their prebiotic effect and is an important source of energy in infants (Mills et al., 2011). Genomic analysis of probiotics from infants also has revealed specific genetic loci related to milk oligosaccharide import and processing, suggesting coevolution between the human host, milk oligosaccharide, and the microbes they enrich (Chichlowski et al., 2011). We also found plentiful probiotics in squab milk (Figure 1C), which are able to consume human milk oligosaccharides (Ward et al., 2006; Thongaram et al., 2017). Therefore, the high abundance of carbohydrate metabolism in squab milk echoed with the presence of potential probiotics, and also implied that the existence of milk microbiota could assist the host by metabolizing nutrients (Ballini et al., 2019). Taken together, our studies suggested that parent pigeons help their offspring grow by transferring the microbiota *via* pigeon milk.

There were abundant *Lactobacillus* and *Bifidobacterium* in squab milk (Figure 1C), which implied that they could be important probiotics associated with growth and development of individuals of squabs. In animals, oral administration of *Bifidobacterium* or *Lactobacillus* has had useful effects in newborn calves and piglets, including improved body weight gain, feed conversion, and fecal condition (Abe et al., 1995). *Lactobacillus* and *Bifidobacteria* can be detected in breast milk after oral supplementation in the mother and in almost all infants after oral supplementation during the first year of life, as well as occasionally in many untreated infants (Abrahamsson et al., 2009; Fernandez et al., 2013). When the squabs grow older, pigeon milk is mixed with grains soaked in the crop of the parents and is gradually replaced by grains only (Vandeputte-Poma, 1980). The existence of *Lactobacillus* and *Bifidobacteria* may be related to the changes of pigeon milk composition so as to protect gastrointestinal tract health of squabs (Figure 2A). It has been discovered that live *Lactobacillus* strains could enhance the barrier function of naïve epithelial cells which are not exposed to any pathogen and alleviate the diarrhea in mice (Resta-Lenert and Barrett, 2003; Wang et al., 2019). Notably, we also detected plentiful functional pathways, including butirosin and neomycin biosynthesis, biosynthesis of vancomycin group antibiotics, dioxin degradation, and xylene degradation in squab milk (Supplementary Table 5). This may indicate that the milk microbiota were involved in the immune system of squabs. Immune-modulating research has suggested that *Lactobacillus* and *Bifidobacterium* show a genus-specific ability to modulate *in vitro* innate immunity, antimicrobial activity against gut pathogens, and reducing colitis and inflammation (Liévin-Le and Servin, 2014; Luongo et al., 2017; Inchingolo et al., 2019). Moderate prenatal stress was sufficient to decrease the numbers of *Lactobacillus* and *Bifidobacterium* in newborn infant monkeys. This alteration could result in heightened susceptibility to infection and suggest a mechanism for some effects of maternal pregnancy conditions on infant health (Bailey et al., 2004). Since a mass of antibiotics biosynthesis pathways was discovered in squab milk (Supplementary Table 5), in agreement with the fact that probiotics are involved in immune system to prevent disease infections (Rosenberg et al., 2016). According to previous report, squabs are easily died or fail to thrive if they fed a nutritional replacement of pigeon milk (Guareschi, 1936). Therefore, it is reasonable to presume that the probiotics was an essential factor in the growth and development of squabs. During pigeon breeding, adding probiotics and changing the proportion of probiotics in artificial pigeon milk with the development stages may improve the survival rate of squabs and promote the production performance of pigeon.

## Conclusion

This study investigates the microbial composition and function in pigeon milk and pigeon intestines. We found abundant microbiota in pigeon milk, which are dominated by the phylum of *Firmicutes* and the genus of *Lactobacillus* and *Bifidobacterium*. The squab milk microbial abundance changes dynamically with growth and development stages, and also related to the changes of microbiota in parent pigeons among different developmental stages. Moreover, microbiota can be transmitted from parents to squabs by pigeon milk. The preponderant genera of parent pigeon milk, such as *Lactobacillus*, are also accounted for a considerable proportion in squab milk. In addition, the intestinal microbial structure of pigeon was affected by the pigeon milk microbiota. Our results indicates that microbiota play an important role in squabs and can be transmitted from parent pigeons to squabs by pigeon milk, and also remind us to consider adding probiotics to the artificial pigeon milk to promote the development of the pigeon industry.

## Data Availability Statement

The datasets presented in this study can be found in online repositories. The names of the repository/repositories and accession number(s) can be found at: https://www.mg-rast.org/mgmain.html?mgpage=project&project=mgp93364, pigeon-milk-microbiota (mgp93364).

## Ethics Statement

The animal study was reviewed and approved by The Institute for Laboratory Animal Research (ILAR) Guide for Care and Use of Laboratory Animals in Shanghai Jiao Tong University.

## Author Contributions

JD wrote the manuscript. JD, NL, and HM conceived and designed the experimental procedure. JD, NL, YZ, and LY collected samples and extracted DNA. JD performed statistical analysis and data processing. HZ, KX, CH, CQ, CT, and LW coordinated sample collection and supervised the study. All authors read, commented and approved the final manuscript.

### Conflict of Interest

CT was employed by Shanghai Xinrong Big Emperor Pigeon Breeding Professional Cooperation.

The remaining authors declare that the research was conducted in the absence of any commercial or financial relationships that could be construed as a potential conflict of interest.

## Footnotes

^1^https://www.mg-rast.org/linkin.cgi?project=mgp93364

^2^http://qiime.org/index.html
