# Corrigendum: The Composition and Function of Pigeon Milk Microbiota Transmitted From Parent Pigeons to Squabs

**DOI:** 10.3389/fmicb.2021.641828

**Published:** 2021-02-18

**Authors:** Jinmei Ding, Nan Liao, Yuming Zheng, Lingyu Yang, Hao Zhou, Ke Xu, Chengxiao Han, Huaixi Luo, Chao Qin, Chunhong Tang, Longxing Wei, He Meng

**Affiliations:** ^1^Shanghai Key Laboratory of Veterinary Biotechnology, Department of Animal Science, School of Agriculture and Biology, Shanghai Jiao Tong University, Shanghai, China; ^2^Shanghai Xinrong Big Emperor Pigeon Breeding Professional Cooperation, Shanghai, China; ^3^Fengxian District Animal Disease Prevention and Control Center, Shanghai, China

**Keywords:** pigeon milk, microbiota, squabs, parent pigeons, composition, function, transmitted

In the original article, there was an error. A portion of the text in the introduction was reproduced from another article, this has now been rephrased and appropriately attributed. A correction has been made to the second paragraph of the **Introduction**, paragraph two, the corrected paragraph appears below:

There is a symbiotic relationship between microbiota and their hosts (Rees et al., 2018; Dietz et al., 2019). The main benefit of microbes was to obtain a relatively stable habitat and adequate food source (Kohl, 2012; McFall-Ngai et al., 2013). Meanwhile, microbes play an important role in many aspects of host physiology, including nutrition, metabolism, and intestinal homeostasis (Walker et al., 2017). Early colonization of microbiota can have long-standing consequences on host such as determining the production of essential metabolites which facilitate postnatal development and enhance immune function (Lee and Mazmanian, 2010; Funkhouser and Bordenstein, 2013; Gensollen et al., 2016; Stinson et al., 2017). Neonates of mammals can acquire maternal microbiota through the placenta, amniotic fluid, vagina, and breast milk (Digiulio et al., 2008; Satokari et al., 2009; Albesharat et al., 2011; Stout et al., 2013; Aagaard et al., 2014). The prenatal exposure is an important step in modulating the embryonic development and the maturation of immune system (Nylund et al., 2014). Fetuses are highly susceptible to disease infections, not only because their immature immune system is less capable of generating adaptive immune effectors, such as antibodies, but also because they lack diverse commensal microbiota that can antagonize pathogens independently of host responses (Basha et al., 2014; Simon et al., 2015; Zheng et al., 2020). Although the chicken embryo is isolated from the mother, the core microbial colonizers of maternal hens can be transmitted to the embryos in the process of fertilization and egg formation in the oviduct (Ding et al., 2017). Likewise, prenatal bacteria transfer may occur in other birds. The relatively high percentage of shared operational taxonomic units (OTUs) between neonates and females is a strong indication that neonates of rock pigeons obtain bacteria through prenatal transfer (Dietz et al., 2019). Research has shown that lactobacilli is important in maintaining a healthy microbial balance in the chicken crop (Fuller, 1977), but as regard to crop secretions, it is not known the pigeon milk microbial composition and function, and whether these microbes can be transmitted from parent pigeons to squabs.

The authors apologize for error and state that this does not change the scientific conclusions of the article in any way. The original article has been updated.
