# Supplementary material for: The Composition and Function of Pigeon Milk Microbiota Transmitted From Parent Pigeons to Squabs
Source: Front Microbiol. 2020 Aug 4;11:1789. doi: 10.3389/fmicb.2020.01789 (PMC7417789; doi:10.3389/fmicb.2020.01789)
Supplement: Supplementary file 1 [file Data_Sheet_1.DOCX]

Supplementary Material

# Supplementary Figures and Tables

## Supplementary Figures

##
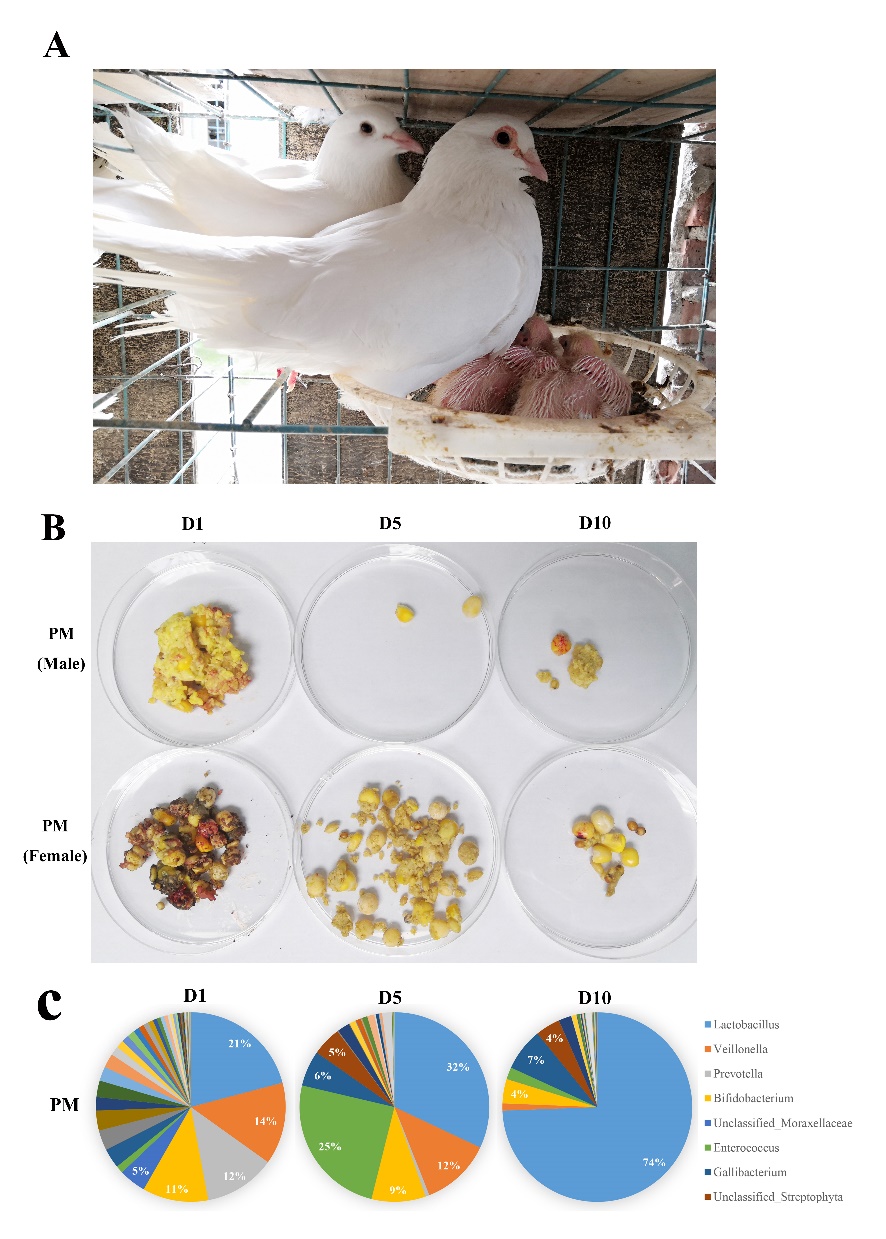


## Supplementary Figure 1. (A) The parent pigeons and their squabs. (B) The morphology of parent pigeon milk after squab hatching at 1-day (D1), 5-day (D5), and 10-day (D10). (C) Distribution of the parent pigeon milk microbiota among different developmental stages at the genus level. Only major taxonomic groups are shown.


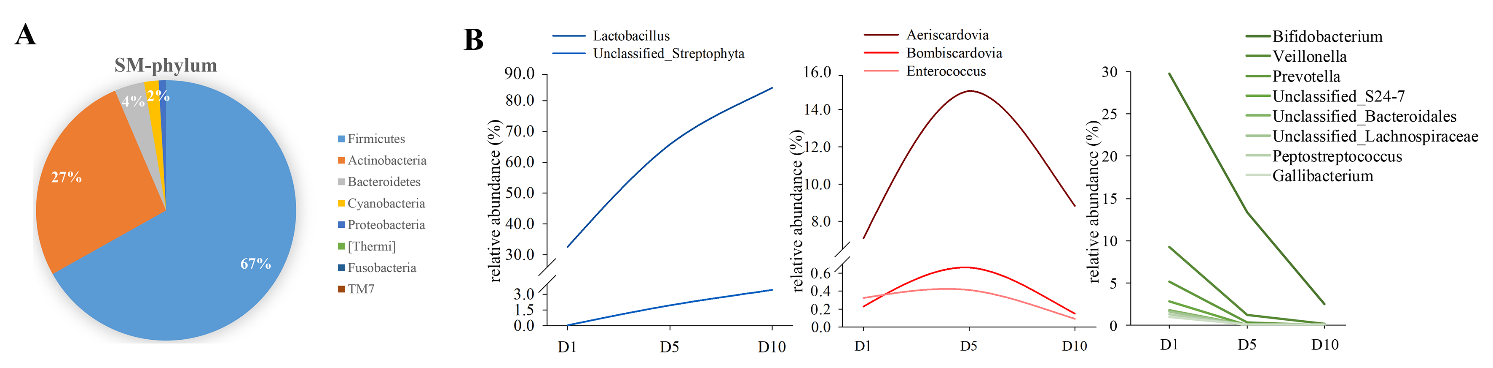


**Supplementary Figure 2.** (A) The composition of squab milk microbiota at the phylum levels. (B) Dynamic distribution of squab milk microbiota at D1, D5, and D10, including the major increasing, decreasing, and the tendency to increase and then decrease microbiota.


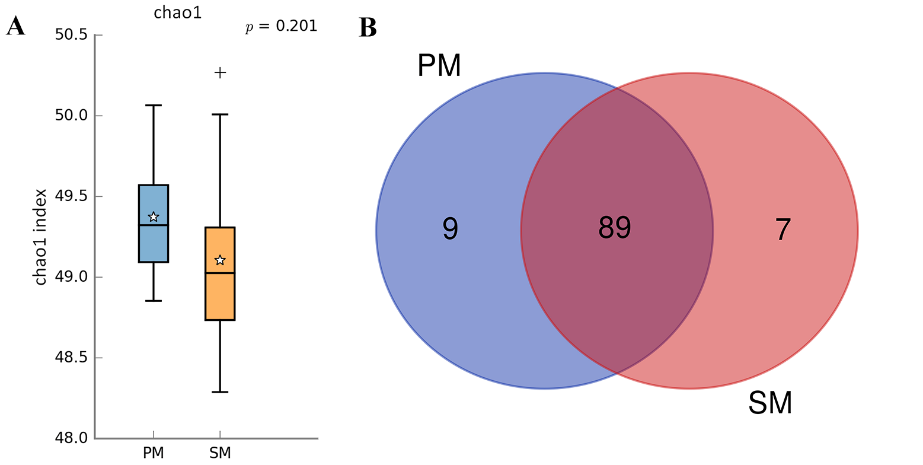


**Supplementary Figure 3.** (A) Alpha diversity analysis by chao1 index for the microbiota of PM and SM. (B) Venn diagram showing the genera shared between PM and SM.


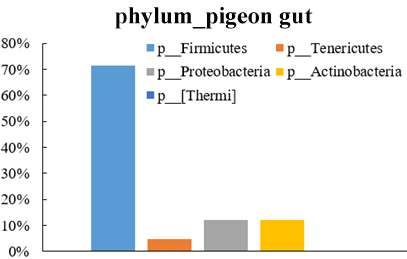


**Supplementary Figure 4.** Distribution of pigeon gut microbiota at the phylum levels.


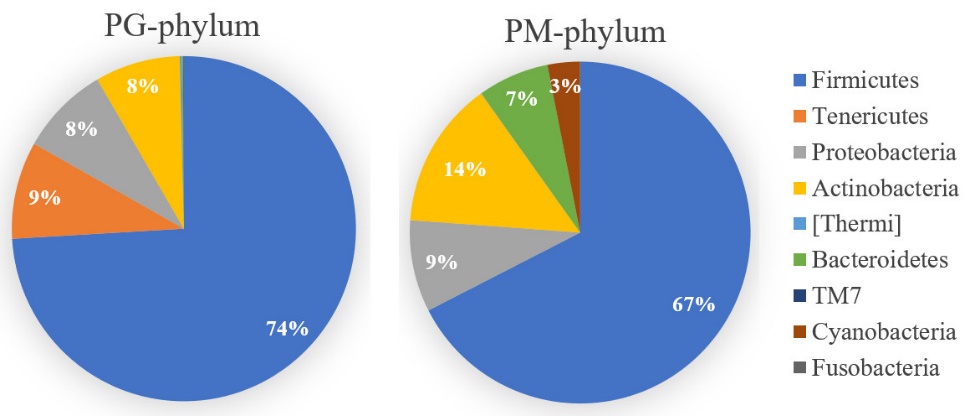


**Supplementary Figure 5.** The comparison of microbiota for PG and PM at the phylum levels.


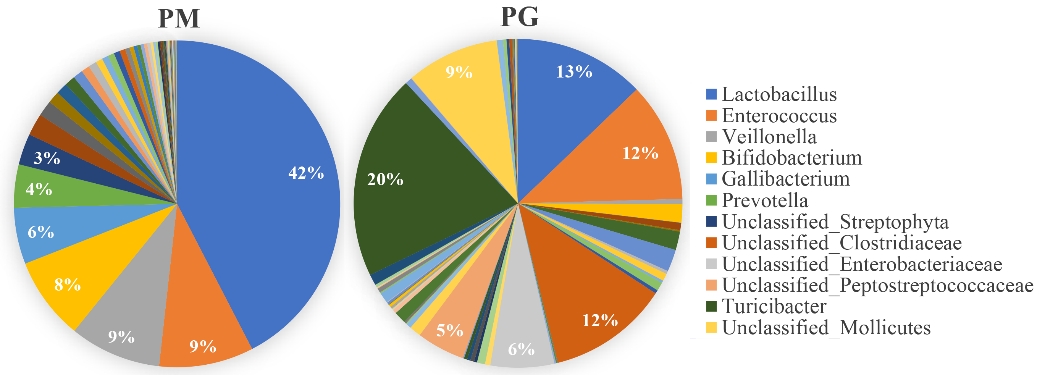


**Supplementary Figure 6.** The comparison of microbiota for PG and PM at the genus levels.

## Supplementary Tables

**Supplementary Table 1:** Significantly different microbes of squab milk at three development stages (D1, D5, and D10).

| genus | I | J | mean difference (I-J) | Standard error | p value |
| --- | --- | --- | --- | --- | --- |
| *g__Capnocytophaga* | D1 | D5 | 1.13 | 0.14 | 0.000 |
| *g__Neisseria* | D1 | D5 | 0.87 | 0.12 | 0.000 |
| *g__Capnocytophaga* | D1 | D10 | 1.01 | 0.14 | 0.000 |
| *g__Streptococcus* | D1 | D5 | -0.72 | 0.10 | 0.000 |
| *g__Unclassified_Streptophyta* | D1 | D10 | -2.68 | 0.39 | 0.000 |
| *g__Neisseria* | D1 | D10 | 0.75 | 0.12 | 0.000 |
| *g__Unclassified_Coriobacteriaceae* | D1 | D10 | 1.53 | 0.26 | 0.001 |
| *g__Unclassified_Coriobacteriaceae* | D1 | D5 | 1.52 | 0.26 | 0.001 |
| *g__Unclassified_Lachnospiraceae* | D1 | D10 | 1.68 | 0.30 | 0.001 |
| *g__Unclassified_Lachnospiraceae* | D1 | D5 | 1.58 | 0.30 | 0.002 |
| *g__Unclassified_Streptophyta* | D1 | D5 | -2.02 | 0.39 | 0.002 |
| *g__Conchiformibius* | D1 | D5 | 0.66 | 0.14 | 0.003 |
| *g__Unclassified_[Mogibacteriaceae]* | D1 | D5 | 0.97 | 0.22 | 0.004 |
| *g__Bombiscardovia* | D1 | D5 | -0.98 | 0.22 | 0.005 |
| *g__Unclassified_[Mogibacteriaceae]* | D1 | D10 | 0.94 | 0.22 | 0.006 |
| *g__Leucobacter* | D1 | D10 | -0.69 | 0.17 | 0.007 |
| *g__Lactobacillus* | D1 | D10 | -1.68 | 0.41 | 0.008 |
| *g__Unclassified_Gemellales* | D1 | D10 | -1.00 | 0.25 | 0.008 |
| *g__Isobaculum* | D5 | D10 | 0.91 | 0.22 | 0.008 |
| *g__Veillonella* | D1 | D10 | 0.86 | 0.21 | 0.008 |
| *g__Corynebacterium* | D1 | D10 | -0.64 | 0.16 | 0.009 |
| *g__Staphylococcus* | D1 | D10 | -1.21 | 0.31 | 0.010 |
| *g__Conchiformibius* | D1 | D10 | 0.54 | 0.14 | 0.010 |
| *g__Streptococcus* | D5 | D10 | 0.39 | 0.10 | 0.011 |
| *g__Proteus* | D1 | D10 | -0.61 | 0.16 | 0.011 |
| *g__Unclassified_S24-7* | D1 | D10 | 1.48 | 0.40 | 0.014 |
| *g__Actinomyces* | D5 | D10 | 0.86 | 0.23 | 0.014 |
| *g__Peptostreptococcus* | D1 | D10 | 1.32 | 0.36 | 0.014 |
| *g__Dietzia* | D1 | D5 | -0.47 | 0.13 | 0.014 |
| *g__Atopobium* | D1 | D10 | 1.09 | 0.31 | 0.018 |
| *g__Unclassified_Bacteroidales* | D1 | D10 | 1.35 | 0.38 | 0.018 |
| *g__Bulleidia* | D1 | D10 | 1.21 | 0.35 | 0.019 |
| *g__Brachybacterium* | D1 | D5 | -0.84 | 0.24 | 0.019 |
| *g__Gallibacterium* | D1 | D5 | 0.47 | 0.13 | 0.020 |
| *g__Unclassified_Clostridiaceae* | D1 | D10 | 1.04 | 0.30 | 0.020 |
| *g__Isobaculum* | D1 | D5 | -0.77 | 0.22 | 0.021 |
| *g__Bacillus* | D1 | D10 | -0.51 | 0.15 | 0.023 |
| *g__Streptococcus* | D1 | D10 | -0.33 | 0.10 | 0.024 |
| *g__Sharpea* | D1 | D5 | 0.87 | 0.26 | 0.025 |
| *g__Unclassified_Clostridiaceae* | D1 | D5 | 0.99 | 0.30 | 0.025 |
| *g__Prevotella* | D1 | D10 | 1.15 | 0.35 | 0.027 |
| *g__Corynebacterium* | D1 | D5 | -0.52 | 0.16 | 0.028 |
| *g__Peptococcus* | D1 | D5 | 0.65 | 0.20 | 0.029 |
| *g__Unclassified_Neisseriaceae* | D5 | D10 | -0.49 | 0.15 | 0.030 |
| *g__Unclassified_Pasteurellaceae* | D1 | D10 | -0.86 | 0.27 | 0.031 |
| *g__Unclassified_Sphingobacteriaceae* | D1 | D10 | -0.69 | 0.22 | 0.033 |
| *g__Brevibacterium* | D1 | D10 | -0.66 | 0.21 | 0.035 |
| *g__Unclassified_Bacteroidales* | D1 | D5 | 1.17 | 0.38 | 0.038 |
| *g__Sutterella* | D1 | D10 | 1.07 | 0.35 | 0.038 |
| *g__Rothia* | D1 | D5 | -0.72 | 0.24 | 0.039 |
| *g__Sutterella* | D1 | D5 | 1.06 | 0.35 | 0.041 |
| *g__Staphylococcus* | D1 | D5 | -0.92 | 0.31 | 0.044 |
| *g__Actinomyces* | D1 | D10 | 0.69 | 0.23 | 0.044 |
| *g__Unclassified_Streptococcaceae* | D1 | D5 | -0.48 | 0.16 | 0.045 |

**Supplementary Table 2:** The third level KEGG metabolic pathways of squab milk microbiota.

| KEGG_Pathways_level3 | relative abundance | KEGG_Pathways_level3 | relative abundance |
| --- | --- | --- | --- |
| Transporters | 7.56% | Glutamatergic synapse | 0.11% |
| DNA repair and recombination proteins | 3.61% | Xylene degradation | 0.10% |
| ABC transporters | 3.42% | Plant-pathogen interaction | 0.10% |
| General function prediction only | 3.34% | Tetracycline biosynthesis | 0.10% |
| Ribosome | 3.13% | Limonene and pinene degradation | 0.10% |
| Purine metabolism | 2.93% | Phenylpropanoid biosynthesis | 0.09% |
| Pyrimidine metabolism | 2.22% | Novobiocin biosynthesis | 0.09% |
| Peptidases | 2.09% | Ascorbate and aldarate metabolism | 0.09% |
| Chromosome | 1.76% | Bacterial chemotaxis | 0.09% |
| Ribosome Biogenesis | 1.64% | Phenylalanine metabolism | 0.09% |
| Amino sugar and nucleotide sugar metabolism | 1.63% | Adipocytokine signaling pathway | 0.09% |
| Aminoacyl-tRNA biosynthesis | 1.61% | Sporulation | 0.08% |
| Transcription factors | 1.60% | Ribosome biogenesis in eukaryotes | 0.08% |
| Amino acid related enzymes | 1.59% | Nucleotide metabolism | 0.08% |
| Glycolysis / Gluconeogenesis | 1.54% | Primary immunodeficiency | 0.08% |
| DNA replication proteins | 1.42% | Bisphenol degradation | 0.07% |
| Function unknown | 1.39% | Lipopolysaccharide biosynthesis | 0.07% |
| Homologous recombination | 1.19% | beta-Alanine metabolism | 0.07% |
| Secretion system | 1.18% | Synthesis and degradation of ketone bodies | 0.07% |
| Others | 1.16% | Ethylbenzene degradation | 0.07% |
| Starch and sucrose metabolism | 1.12% | Biosynthesis of vancomycin group antibiotics | 0.07% |
| Other ion-coupled transporters | 1.12% | Tropane, piperidine and pyridine alkaloid biosynthesis | 0.07% |
| Alanine, aspartate and glutamate metabolism | 1.06% | Drug metabolism - cytochrome P450 | 0.07% |
| Methane metabolism | 1.05% | Metabolism of xenobiotics by cytochrome P450 | 0.07% |
| Two-component system | 1.01% | Lysine degradation | 0.07% |
| Chaperones and folding catalysts | 1.00% | Ion channels | 0.07% |
| Pyruvate metabolism | 1.00% | Biotin metabolism | 0.07% |
| Replication, recombination and repair proteins | 0.99% | Linoleic acid metabolism | 0.06% |
| Cysteine and methionine metabolism | 0.99% | Glycosphingolipid biosynthesis - globo series | 0.06% |
| Mismatch repair | 0.97% | Type II diabetes mellitus | 0.06% |
| Fructose and mannose metabolism | 0.96% | Type I diabetes mellitus | 0.06% |
| Peptidoglycan biosynthesis | 0.95% | Butirosin and neomycin biosynthesis | 0.06% |
| Pentose phosphate pathway | 0.93% | Alzheimer's disease | 0.06% |
| Translation proteins | 0.92% | Zeatin biosynthesis | 0.06% |
| Phosphotransferase system (PTS) | 0.91% | Vibrio cholerae pathogenic cycle | 0.06% |
| Oxidative phosphorylation | 0.89% | Retinol metabolism | 0.06% |
| Lysine biosynthesis | 0.86% | Primary bile acid biosynthesis | 0.05% |
| DNA replication | 0.86% | Cellular antigens | 0.05% |
| Arginine and proline metabolism | 0.81% | Secondary bile acid biosynthesis | 0.05% |
| Carbon fixation pathways in prokaryotes | 0.80% | Insulin signaling pathway | 0.05% |
| Galactose metabolism | 0.79% | Lipoic acid metabolism | 0.05% |
| Transcription machinery | 0.72% | Biosynthesis of ansamycins | 0.05% |
| Glycine, serine and threonine metabolism | 0.71% | Photosynthesis - antenna proteins | 0.04% |
| Terpenoid backbone biosynthesis | 0.70% | Lysosome | 0.04% |
| Photosynthesis proteins | 0.67% | Protein processing in endoplasmic reticulum | 0.04% |
| Protein export | 0.66% | Epithelial cell signaling in Helicobacter pylori infection | 0.03% |
| Translation factors | 0.65% | Cell division | 0.03% |
| Carbon fixation in photosynthetic organisms | 0.64% | Pathways in cancer | 0.03% |
| Photosynthesis | 0.62% | Phosphonate and phosphinate metabolism | 0.03% |
| One carbon pool by folate | 0.61% | Proteasome | 0.03% |
| Butanoate metabolism | 0.60% | Renal cell carcinoma | 0.03% |
| Protein folding and associated processing | 0.60% | beta-Lactam resistance | 0.03% |
| Cell cycle - Caulobacter | 0.59% | Penicillin and cephalosporin biosynthesis | 0.03% |
| Signal transduction mechanisms | 0.59% | Proximal tubule bicarbonate reclamation | 0.03% |
| Nitrogen metabolism | 0.58% | Isoquinoline alkaloid biosynthesis | 0.03% |
| Thiamine metabolism | 0.55% | Carotenoid biosynthesis | 0.02% |
| Lipid biosynthesis proteins | 0.54% | Carbohydrate digestion and absorption | 0.02% |
| Glycerophospholipid metabolism | 0.54% | Ubiquitin system | 0.02% |
| Bacterial secretion system | 0.54% | N-Glycan biosynthesis | 0.02% |
| Valine, leucine and isoleucine biosynthesis | 0.53% | Glycosaminoglycan degradation | 0.02% |
| Nucleotide excision repair | 0.53% | Flagellar assembly | 0.02% |
| RNA degradation | 0.52% | Chlorocyclohexane and chlorobenzene degradation | 0.02% |
| Base excision repair | 0.52% | Caprolactam degradation | 0.01% |
| Nicotinate and nicotinamide metabolism | 0.51% | Nitrotoluene degradation | 0.01% |
| Phenylalanine, tyrosine and tryptophan biosynthesis | 0.50% | Biosynthesis and biodegradation of secondary metabolites | 0.01% |
| Propanoate metabolism | 0.49% | Glycosphingolipid biosynthesis - ganglio series | 0.01% |
| Pantothenate and CoA biosynthesis | 0.49% | African trypanosomiasis | 0.01% |
| Tyrosine metabolism | 0.48% | Chagas disease (American trypanosomiasis) | 0.01% |
| Prenyltransferases | 0.47% | Protein digestion and absorption | 0.01% |
| Citrate cycle (TCA cycle) | 0.45% | Apoptosis | 0.01% |
| Energy metabolism | 0.42% | Transcription related proteins | 0.01% |
| Selenocompound metabolism | 0.42% | Meiosis - yeast | 0.01% |
| Glycosyltransferases | 0.41% | Geraniol degradation | 0.01% |
| Glycerolipid metabolism | 0.41% | Glycan biosynthesis and metabolism | 0.01% |
| Fatty acid biosynthesis | 0.41% | Huntington's disease | 0.01% |
| Histidine metabolism | 0.39% | Stilbenoid, diarylheptanoid and gingerol biosynthesis | 0.01% |
| Porphyrin and chlorophyll metabolism | 0.39% | Atrazine degradation | 0.01% |
| Folate biosynthesis | 0.37% | Mineral absorption | 0.01% |
| Cytoskeleton proteins | 0.36% | Flavonoid biosynthesis | 0.01% |
| Streptomycin biosynthesis | 0.35% | MAPK signaling pathway - yeast | 0.01% |
| Benzoate degradation | 0.31% | RIG-I-like receptor signaling pathway | 0.01% |
| Glutathione metabolism | 0.31% | Arachidonic acid metabolism | 0.01% |
| Drug metabolism - other enzymes | 0.31% | Biosynthesis of siderophore group nonribosomal peptides | 0.01% |
| Membrane and intracellular structural molecules | 0.31% | Pertussis | 0.01% |
| Fatty acid metabolism | 0.29% | Fluorobenzoate degradation | 0.01% |
| Pentose and glucuronate interconversions | 0.28% | Steroid biosynthesis | 0.01% |
| Protein kinases | 0.27% | D-Arginine and D-ornithine metabolism | 0.00% |
| RNA polymerase | 0.27% | Amoebiasis | 0.00% |
| Sulfur metabolism | 0.26% | Styrene degradation | 0.00% |
| Ubiquinone and other terpenoid-quinone biosynthesis | 0.26% | Antigen processing and presentation | 0.00% |
| Glyoxylate and dicarboxylate metabolism | 0.26% | NOD-like receptor signaling pathway | 0.00% |
| Chloroalkane and chloroalkene degradation | 0.24% | Progesterone-mediated oocyte maturation | 0.00% |
| Riboflavin metabolism | 0.23% | Prostate cancer | 0.00% |
| Sulfur relay system | 0.23% | Flavone and flavonol biosynthesis | 0.00% |
| Naphthalene degradation | 0.22% | Electron transfer carriers | 0.00% |
| Pores ion channels | 0.21% | alpha-Linolenic acid metabolism | 0.00% |
| D-Glutamine and D-glutamate metabolism | 0.20% | Steroid hormone biosynthesis | 0.00% |
| Tuberculosis | 0.20% | Bacterial invasion of epithelial cells | 0.00% |
| Cyanoamino acid metabolism | 0.20% | Calcium signaling pathway | 0.00% |
| Valine, leucine and isoleucine degradation | 0.20% | Germination | 0.00% |
| Restriction enzyme | 0.19% | Amyotrophic lateral sclerosis (ALS) | 0.00% |
| Bacterial motility proteins | 0.19% | Parkinson's disease | 0.00% |
| D-Alanine metabolism | 0.18% | Non-homologous end-joining | 0.00% |
| Polyketide sugar unit biosynthesis | 0.18% | Cardiac muscle contraction | 0.00% |
| Bacterial toxins | 0.17% | Prion diseases | 0.00% |
| C5-Branched dibasic acid metabolism | 0.17% | Bladder cancer | 0.00% |
| Peroxisome | 0.17% | Basal transcription factors | 0.00% |
| Cell motility and secretion | 0.17% | Ether lipid metabolism | 0.00% |
| Vitamin B6 metabolism | 0.17% | p53 signaling pathway | 0.00% |
| Sphingolipid metabolism | 0.16% | Colorectal cancer | 0.00% |
| Polycyclic aromatic hydrocarbon degradation | 0.16% | Influenza A | 0.00% |
| Aminobenzoate degradation | 0.16% | Small cell lung cancer | 0.00% |
| Lipid metabolism | 0.16% | Toxoplasmosis | 0.00% |
| Carbohydrate metabolism | 0.16% | Viral myocarditis | 0.00% |
| Taurine and hypotaurine metabolism | 0.15% | Circadian rhythm - plant | 0.00% |
| Other glycan degradation | 0.15% | Systemic lupus erythematosus | 0.00% |
| Inorganic ion transport and metabolism | 0.15% | Pathogenic Escherichia coli infection | 0.00% |
| Toluene degradation | 0.14% | Bile secretion | 0.00% |
| RNA transport | 0.14% | Renin-angiotensin system | 0.00% |
| Staphylococcus aureus infection | 0.13% | Caffeine metabolism | 0.00% |
| Lipopolysaccharide biosynthesis proteins | 0.13% | 1,1,1-Trichloro-2,2-bis(4-chlorophenyl)ethane (DDT) degradation | 0.00% |
| Other transporters | 0.13% | Shigellosis | 0.00% |
| PPAR signaling pathway | 0.13% | Isoflavonoid biosynthesis | 0.00% |
| Dioxin degradation | 0.13% | Endocytosis | 0.00% |
| Biosynthesis of unsaturated fatty acids | 0.12% | Fc gamma R-mediated phagocytosis | 0.00% |
| Tryptophan metabolism | 0.12% | GnRH signaling pathway | 0.00% |
| Amino acid metabolism | 0.12% | Hypertrophic cardiomyopathy (HCM) | 0.00% |
| Inositol phosphate metabolism | 0.12% | Various types of N-glycan biosynthesis | 0.00% |
| Metabolism of cofactors and vitamins | 0.12% | Biosynthesis of type II polyketide products | 0.00% |
| Phosphatidylinositol signaling system | 0.11% |  |  |

**Supplementary Table 3:** Relative abundance of microbiota at the genus level for PG and SG (p<0.05).

| Genus | PG-mean | SG-mean | p-value |
| --- | --- | --- | --- |
| *g__Turicibacter* | 2.85 | 1.45 | 0.002 |
| *g__Lactobacillus* | 2.87 | 4.57 | 0.002 |
| *g__Enterococcus* | 2.56 | 1.54 | 0.029 |
| *g__Bifidobacterium* | 1.57 | 2.58 | 0.039 |
| *g__Trichococcus* | 1.58 | 0.40 | 0.021 |
| *g__Unclassified_Clostridiales* | 1.23 | 0.59 | 0.043 |
| *g__Unclassified_Enterococcaceae* | 0.90 | -0.26 | 0.027 |
| *g__Pediococcus* | -0.63 | -0.14 | 0.033 |

**Supplementary Table 4:** Relative abundance of microbiota at the genus level for PG and PM (p<0.05).

| Taxon | PM-mean | PG-mean | Relative fold change | p value |
| --- | --- | --- | --- | --- |
| *g__Gallibacterium* | 2.43 | -0.43 | 7.27 | 0.04 |
| *g__Veillonella* | 2.66 | 0.64 | 4.05 | 0.02 |
| *g__Lactobacillus* | 3.94 | 3.07 | 1.83 | 0.01 |
| *g__Pediococcus* | -0.01 | -0.58 | 1.48 | 0.01 |
| *g__Unclassified_Pasteurellaceae* | -0.13 | -0.58 | 1.36 | 0.01 |
| *g__Granulicatella* | -0.21 | -0.58 | 1.29 | 0.05 |
| *g__Unclassified_Burkholderiales* | -0.56 | 0.1 | -1.58 | 0.03 |
| *g__Unclassified_Clostridiales* | -0.26 | 1.38 | -3.12 | 0.05 |
| *g__Trichococcus* | -0.01 | 1.73 | -3.34 | 0.02 |
| *g__Clostridium* | -0.46 | 1.4 | -3.62 | 0.02 |
| *g__Unclassified_Clostridiaceae* | 0.53 | 2.83 | -4.91 | 0.02 |
| *g__Turicibacter* | -0.44 | 3.05 | -11.25 | 0.01 |

**Supplementary Table 5:** Significant differences in microbial metabolic pathways for PM and SM.

| KEGG level2 | KEGG level3 | PM-mean | SM-mean | p-value |
| --- | --- | --- | --- | --- |
| Amino Acid Metabolism | Phenylalanine metabolism | 167.26 | 161.46 | 0.02 |
| Cell Motility | Flagellar assembly | 144.75 | 128.68 | 0.002 |
| Glycan Biosynthesis and Metabolism | Lipopolysaccharide biosynthesis | 170.32 | 157.39 | 0.01 |
| Glycan Biosynthesis and Metabolism | Lipopolysaccharide biosynthesis proteins | 177.70 | 168.07 | 0.01 |
| Infectious Diseases | Pertussis | 130.34 | 111.59 | 0.01 |
| Lipid Metabolism | Ether lipid metabolism | 72.87 | 48.29 | 0.03 |
| Metabolism | Glycan biosynthesis and metabolism | 135.18 | 118.20 | 0.01 |
| Metabolism | Biosynthesis and biodegradation of secondary metabolites | 139.07 | 127.77 | 0.04 |
| Metabolism of Cofactors and Vitamins | Biotin metabolism | 163.35 | 155.99 | 0.02 |
| Metabolism of Other Amino Acids | Phosphonate and phosphinate metabolism | 149.25 | 141.65 | 0.03 |
| Metabolism of Terpenoids and Polyketides | Biosynthesis of siderophore group nonribosomal peptides | 131.32 | 114.10 | 0.01 |
| Metabolism of Terpenoids and Polyketides | Geraniol degradation | 140.40 | 123.34 | 0.02 |
| Neurodegenerative Diseases | Prion diseases | 98.15 | 61.91 | 0.0002 |
| Neurodegenerative Diseases | Amyotrophic lateral sclerosis (ALS) | 118.16 | 87.05 | 0.0001 |
| Neurodegenerative Diseases | Huntington's disease | 136.68 | 118.97 | 0.01 |
| Replication and Repair | Non-homologous end-joining | 103.17 | 74.85 | 0.001 |
| Signal Transduction | MAPK signaling pathway - yeast | 131.02 | 112.89 | 0.01 |
| Transcription | Basal transcription factors | 72.91 | 49.64 | 0.01 |
| Xenobiotics Biodegradation and Metabolism | Caprolactam degradation | 141.65 | 129.14 | 0.02 |
| Amino Acid Metabolism | Lysine biosynthesis | 197.32 | 201.06 | 0.02 |
| Biosynthesis of Other Secondary Metabolites | Butirosin and neomycin biosynthesis | 149.19 | 155.30 | 0.05 |
| Biosynthesis of Other Secondary Metabolites | Flavone and flavonol biosynthesis | 83.98 | 102.09 | 0.03 |
| Carbohydrate Metabolism | Galactose metabolism | 194.32 | 199.56 | 0.02 |
| Carbohydrate Metabolism | Starch and sucrose metabolism | 200.04 | 205.41 | 0.03 |
| Digestive System | Protein digestion and absorption | 91.93 | 119.43 | 0.04 |
| Enzyme Families | Peptidases | 212.55 | 216.37 | 0.04 |
| Glycan Biosynthesis and Metabolism | Other glycan degradation | 160.63 | 170.37 | 0.01 |
| Immune System Diseases | Primary immunodeficiency | 153.92 | 159.57 | 0.02 |
| Lipid Metabolism | Sphingolipid metabolism | 162.01 | 172.12 | 0.003 |
| Metabolic Diseases | Type I diabetes mellitus | 152.04 | 155.72 | 0.05 |
| Metabolism | Nucleotide metabolism | 154.10 | 159.53 | 0.04 |
| Metabolism of Cofactors and Vitamins | Thiamine metabolism | 189.67 | 193.48 | 0.02 |
| Metabolism of Cofactors and Vitamins | Nicotinate and nicotinamide metabolism | 187.65 | 191.86 | 0.03 |
| Metabolism of Other Amino Acids | D-Alanine metabolism | 170.14 | 174.13 | 0.04 |
| Metabolism of Other Amino Acids | Taurine and hypotaurine metabolism | 166.63 | 171.03 | 0.05 |
| Metabolism of Other Amino Acids | D-Glutamine and D-glutamate metabolism | 171.09 | 175.83 | 0.01 |
| Metabolism of Terpenoids and Polyketides | Zeatin biosynthesis | 150.16 | 154.49 | 0.01 |
| Metabolism of Terpenoids and Polyketides | Biosynthesis of vancomycin group antibiotics | 152.06 | 157.31 | 0.04 |
| Nervous System | Glutamatergic synapse | 159.00 | 165.57 | 0.002 |
| Nucleotide Metabolism | Pyrimidine metabolism | 213.75 | 217.43 | 0.02 |
| Replication and Repair | Mismatch repair | 199.56 | 203.07 | 0.03 |
| Replication and Repair | DNA replication proteins | 206.01 | 209.72 | 0.03 |
| Replication and Repair | DNA repair and recombination proteins | 222.08 | 225.86 | 0.03 |
| Replication and Repair | Homologous recombination | 202.73 | 206.72 | 0.01 |
| Replication and Repair | DNA replication | 196.84 | 200.95 | 0.01 |
| Replication and Repair | Nucleotide excision repair | 187.59 | 192.56 | 0.01 |
| Signaling Molecules and Interaction | Cytoskeleton proteins | 181.36 | 185.89 | 0.03 |
| Signaling Molecules and Interaction | Bacterial toxins | 167.26 | 173.30 | 0.01 |
| Signaling Molecules and Interaction | Cellular antigens | 138.91 | 151.09 | 0.01 |
| Transcription | RNA polymerase | 176.18 | 180.69 | 0.02 |
| Translation | Translation factors | 192.66 | 196.28 | 0.02 |
| Translation | Ribosome | 219.24 | 223.38 | 0.01 |
| Translation | Aminoacyl-tRNA biosynthesis | 207.62 | 211.86 | 0.01 |
| Translation | Ribosome biogenesis in eukaryotes | 154.60 | 159.83 | 0.01 |
| Transport and Catabolism | Lysosome | 135.04 | 146.22 | 0.03 |
| Xenobiotics Biodegradation and Metabolism | Drug metabolism - other enzymes | 180.13 | 183.34 | 0.03 |
| Xenobiotics Biodegradation and Metabolism | Dioxin degradation | 161.08 | 167.46 | 0.03 |
| Xenobiotics Biodegradation and Metabolism | Xylene degradation | 157.21 | 163.99 | 0.04 |
